# Comment by European Alzheimer’s Disease Consortium (EADC) investigators on the negative recommendation of the CHMP on the marketing authorization of donanemab for early Alzheimer’ s disease

**DOI:** 10.1016/j.tjpad.2025.100259

**Published:** 2025-07-09

**Authors:** Frank Jessen, Javier Arbizu, Mercé Boada, Mircea Balasa, Karim Bennys, Marina Boban, Katharina Bürger, Andrea Chincarini, Annachiara Cagnin, Peter Paul De Deyn, Emrah Düzel, Sebastiaan Engelborghs, Michael Ewers, Lieza G. Exalto, Wiesje M. van der Flier, Juan Fortea, Kristian Steen Frederiksen, Giovanni B Frisoni, Lutz Frölich, Alejandro J. Garza-Martinez, Timo Grimmer, Bernard Hanseeuw, Jakub Hort, Adrian Ivanoiu, Patrick G Kehoe, Sean P Kennelly, Silke Kern, Stefan Klöppel, Lenka Krajčovičová, Milica G. Kramberger, Bernadette McGuinness, Patrizia Mecocci, Timo Jan Oberstein, Pierre-Jean Ousset, Claire Paquet, Robert Perneczky, Fabrizio Piazza, Domenico Plantone, Innocenzo Rainero, Guillaume Sacco, Eric Salmon, Isabel Santana, Nikolaos Scarmeas, Anja Schneider, Jonathan M Schott, Eino Solje, Elka Stefanova, Elisabeth Stögmann, Mélanie Strauss, Stanislav Sutovsky, Gunhild Waldemar, Bengt Winblad

**Affiliations:** a Department of Psychiatry, University of Cologne, Medical Faculty, Cologne, Germany; b German Center for Neurodegenerative Diseases (DZNE), Bonn/Cologne, Germany; c Department of Nuclear Medicine, University of Navarre Clinic, Pamplona-Madrid, Spain; d Navarra Institute for Health Research (IdiSNA), Pamplona, Spain; e Centro de Investigación Biomédica en Red en Enfermedades Neurodegenerativas (CIBERNED), Spain; f Ace Alzheimer Center Barcelona, Universitat Internacional de Catalunya, Barcelona, Spain; g Alzheimer’s disease and other cognitive disorders Unit, Hospital Clínic de Barcelona, Fundació Recerca Clínic Barcelona-IDIBAPS, Spain; h Memory Research Ressources Center for Alzheimer’s disease, Cognitive Behavioral Neurology Unit, University Hospital Montpellier, France; i Department of Cognitive Neurology, University Hospital Centre Zagreb, School of Medicine, University of Zagreb, Zagreb, Croatia; j Institute for Stroke and Dementia Research, University Hospital, LMU Munich, Germany; k German Center for Neurodegenerative Diseases (DZNE) Munich, Munich, Germany; l National Institute for Nuclear Physics (INFN), Genova, Italy; m Department of Neuroscience and Padua Neuroscience Center, University of Padua, Italy; n Laboratory of Neurochemistry and Behavior, Experimental Neurobiology unit, Department of Biomedical Sciences, University of Antwerp and Memory Clinic Antwerp, ZAS Antwerp, Belgium; o Institute of Cognitive Neurology and Dementia Research, Univ of Magdeburg, Germany; p German Center for Neurodegenerative Diseases (DZNE) Magdeburg, Germany; q Department of Neurology, Universitair Ziekenhuis Brussel and NEUR Research Group, Center for Neurosciences (C4N), Vrije Universiteit Brussel (VUB), Brussels, Belgium; r Department of Biomedical Sciences, University of Antwerp, Antwerp, Belgium; s Michael Ewers, Institute for Stroke and Dementia Research, University Hospital, Ludwig Maximilian University Munich, Germany; t Department of Neurology, UMC Utrecht Brain Center, University Medical Center Utrecht, Utrecht, Netherlands; u Alzheimer Center Amsterdam, Neurology, Vrije Universiteit Amsterdam, Amsterdam UMC location VUmc, Amsterdam, the Netherlands; v Julius Clinical, Zeist, the Netherlands; w Amsterdam Neuroscience, Neurodegeneration, Amsterdam, the Netherlands; x Epidemiology and Data Science, Vrije Universiteit Amsterdam, Amsterdam UMC location VUmc, Amsterdam, the Netherlands; y Sant Pau Memory Unit, Neurology Department, Institut d’Investigacions Biomèdiques Sant Pau, Hospital de la Santa Creu i Sant Pau, Universitat Autònoma de Barcelona, Barcelona, Spain; z Danish Dementia Research Centre, Copenhagen University hospital -Rigshospitalet, Copenhagen, Denmark; aa Department of Clinical Medicine, University of Copenhagen, Copenhagen, Denmark; ab Memory Center, Geneva University and University Hospitals, Geneva, Switzerland; ac Department of Geriatric Psychiatry, Central Institute of Mental Health, Medical Faculty Mannheim, Heidelberg University, Germany; ad Servicio de Geriatría, Hospital Universitario Ramón y Cajal (IRICYS), Madrid, Spain; ae Department of Psychiatry and Psychotherapy, TUM University Hospital, School of Medicine and Health, Technical University of Munich, Munich, Germany; af Department of Neurology, Cliniques Universitaires Saint-Luc, Institute of Neurosciences, Université catholique de Louvain, Brussels, Belgium; ag Department of Neurology, Charles University, Second Medical Faculty and Motol University Hospital, Prague, Czech Republic; ah Cerebrovascular and Dementia Research Group, Bristol Medical School, University of Bristol, Bristol, UK; ai Tallaght Institute of Memory and Cognition, Tallaght University Hospital, Dublin, Ireland; aj Department of Medical Gerontology, School of Medicine, Trinity College Dublin, Ireland; ak Institute of Neuroscience and Physiology, Sahlgrenska Academy at the University of Gothenburg, Sweden; al Region Västra Götaland, Sahlgrenksa University Hospital, Department of Neuropsychiatry, Gothenburg, Sweden; am University Hospital of Hold Age Psychiatry, University of Bern, Bern, Switzerland; an Department of Neurology, Faculty of Medicine, Masaryk University and Saint Anne´s University Hospital, Brno, Czech Republic; ao Department of Neurology, University Medical Center, Medical faculty, Ljubljana University of Ljubljana, Ljubljana, Slovenia; ap Karolinska Institutet, Department of Neurobiology, Care Sciences and Society (NVS), Division of Clinical Geriatrics, Huddinge, Sweden; aq Centre for Public Health, Institute of Clinical Sciences B, Queen’s University Belfast, UK; ar Division of Gerontology and Geriatrics, Department of Medicine and Surgery, University of Perugia, Italy; as Division of Clinical Geriatrics, NVS Department, Karolinska Institutet Stockholm, Sweden; at Department of Psychiatry and Psychotherapy, Uniklinikum Erlangen, Friedrich-Alexander-Universität Erlangen-Nürnberg (FAU), Erlangen, Germany; au Institut Hospitalo Universitaire HealthAge, Alzheimer's Disease Research and Clinical Center, Toulouse University Hospital, Toulouse, France; av Cognitive Neurology Center, GHU APHP Nord Lariboisière Fernand-Widal, Université de Paris Cité - INSERM 1144, Faculty of Medicine, Paris, France; aw Department of Psychiatry and Psychotherapy, University Hospital, LMU Munich, Munich, Germany; ax Munich Cluster for Systems Neurology (SyNergy), 7 Munich, Germany; ay Ageing Epidemiology (AGE) Research Unit, School of Public Health, Imperial College London, London, UK; az Sheffield Institute for Translational Neuroscience (SITraN), University of Sheffield, Sheffield, UK; ba CAA and AD Translational Research and Biomarkers Laboratory, School of Medicine and Surgery, University of Milano Bicocca, Monza, Italy; bb Department of Medicine, Surgery and Neuroscience, University of Siena, Italy; bc Memory Clinic, Department of Neuroscience “Rita Levi Montalcin”, University of Torino, Italy; bd Université Côte d’Azur, Centre Hospitalier Universitaire de Nice, Clinique Gériatrique du cerveau et du mouvement, Centre Mémoire de Ressources et de Recherche, Nice, France; be Université Côte d'Azur, INSERM, CNRS, IPMC, Equipe AlzPark, Nice, France; bf Memory Clinic, Liege University Hospital, Liege, Belgium; bg Faculty of Medicine,University of Coimbra, Coimbra, Portugal; bh Department of Neurology, Hospital da Universidade de Coimbra, ULS de Coimbra, Coimbra, Portugal; bi 1st department of Neurology, Medical School, National and Kapodistrian University of Athens, Aiginitio Hospital, Athens, Greece; bj Department of Neurology, Columbia University, New York, NY, USA; bk Department of Old Age Psychiatry and Cognitive Disorders, University Hospital Bonn, Bonn, Germany; bl German Center for Neurodegenerative Diseases (DZNE), Bonn, Germany; bm Dementia Research Centre,Queen Square Instiute of Neurology, London, UK; bn UK Dementia Research Institute, UK; bo Institute of Clinical Medicine - Neurology, University of Eastern Finland, Kuopio, Finland; bp Neuro Center - Neurology, Kuopio University Hospital, Kuopio, Finland; bq University of Belgrade, Faculty of Medicine, Serbia; br Department of Neurology, Medical University of Vienna, Austria; bs Université libre de Bruxelles (ULB), Hôpital Universitaire de Bruxelles (H.U.B), CUB Hôpital Érasme, Service de Neurologie, Bruxelles, Belgium; bt 1st Department of Neurology, Faculty of Medicine, Comenius University and University Hospital, Bratislava, Slovakia; bu Danish Dementia Research Centre, Department of Neurology, Copenhagen University Hospital - Rigshospitalet, Denmark; bv Department of Clinical Medicine, University of Copenhagen, Denmark; bw Div of Neurogeriatrics, Dept NVS, Karolinska Institutet, Stockholm and Theme Inflammation & Aging, Karolinska University Hospital, Huddinge, Sweden

On March 27th, 2025 the Committee for Medical Product for Human Use (CHMP) of the European Medicines Agency (EMA) recommended the refusal of the marketing authorization of donanemab for the treatment of early Alzheimer disease (AD), while on November 14th 2024 the same committee gave a positive recommendation for lecanemab for the same indication. Lecanemab was fully approved by the European Commission on the 15th of April 2025.

The rationale for the CHMP’s opposing recommendation remains unclear. Lecanemab and donanemab are comparable in efficacy. In fact, the donanemab vs. placebo difference at 18 months was 0.67 points on the Clinical Dementia Rating Sum of Boxes (CDR-SB) in the phase 3 clinical study TRAILBLAZER-ALZ 2, while it was 0.45 points in the lecanemab phase 3 study CLARITY-AD [1,2].

The CHMP argued that the risks of taking donanemab would not outweigh its benefit even after restriction to non-carriers of the Apolipoprotein Eε4 (*APOE* ε4) allele, who are at lowest risk of side effects. We acknowledge that the overall Amyloid Related Imaging Abnormality (ARIA) rate in TRAILBLAZER-ALZ 2 was higher than in CLARITY-AD [1,2]. However, given that ARIA are mostly asymptomatic and, if symptomatic, mostly of mild to moderate severity and transient, the very few cases of severe ARIA-E and macrohemorrhages with fatal outcome are the primary safety concerns. Of a total of 557 *APOE* ε4 non-carriers in TRAILBLAZER-ALZ 2, one died of an ARIA-related intracerebral hemorrhage [1]. No further deaths in *APOE* ε4 non-carriers in relation to ARIA have been reported neither in the initial TRAILBLAZER-ALZ study [3], nor in the TRAILBLAZER-ALZ 2 extension study [4], or in the recent TRAILBLAZER-ALZ 6 study [5]. The pooled number of *APOE* ε4 non-carriers exposed to donanemab treatment across these studies is around 840, yielding a percentage of 0.1 % fatal cases (*n* = 1). This single case had superficial siderosis at baseline [1], which was not an exclusion criterion in TRAILBLAZER-ALZ 2, but should arguably be one in the label of donanemab as it is in the case of lecanemab. Hence, with baseline superficial siderosis and *APOE* ε4 carrier status as exclusion criteria, there would have been most likely no treatment-related death in TRAILBLAZER-ALZ 2 and there was none in any of the other studies of the TRAILBLAZER-ALZ program.

In the TRAILBLAZER-ALZ 6 study, modified titration schemes were tested regarding ARIA frequency. One scheme with a lower starting dose but dose equivalence after three months showed similar pharmacokinetics to the standard dosing scheme of TRAILBLAZER-ALZ 2 in combination with lower ARIA rates in both, *APOE* ε4 non-carriers and *APOE* ε4 carrier [5]. TRAILBLAZER-ALZ 6 was designed without a placebo group so that efficacy against placebo cannot be derived from that study. The modified titration scheme of TRAILBLAZER-ALZ 6 is already used in treatment centers in the United States and is under regulatory review by the United States Food and Drug Administration (FDA) and other regulatory bodies.

In addition, the CHMP concluded that the effect of donanemab on the primary outcome (integrated Alzheimer’s Disease Rating Scale, iADRS) was smaller in the *APOE* ε4 non-carrier subgroup than in the total group. In contrast, however, the data reported in the TRAILBLAZER-ALZ 2 supplement show greater efficacy of the donanemab in the *APOE* ε4 non-carrier group [1]. It remains unclear on which analyses the CHMP conclusion is based.

The recommendation against donanemab also does not consider the benefits for patients and care partners related to the lower infusion frequency of donanemab compared with lecanemab (four weekly vs two weekly) and of the stopping rules (after 18 months or earlier after amyloid clearance with donanemab vs. continuous treatment with lecanemab). According to our clinical experience, the benefits for patients and care partners and important aspects in treatment decisions are not solely related to efficacy and safety, but also - to a significant extend - to lower treatment-related burden. Finally, having a choice between treatments not only benefits patients and care partners, but also heath care systems, for example regarding pricing, and it serves the prospective development of treatments in real world care.

In summary, the authors consider the discrepancy between the positive recommendation for lecanemab and the negative recommendation against donanemab arbitrary, not consistent with the scientific evidence, and not to the benefit of patients and care partners. We hope that the CHMP reconsiders this decision and eventually provides a positive opinion in alignment with lecanemab, which may follow the same safety measures, including a Controlled Access Programme (CAP) and a Post Authorization Safety Study (PASS). We are strongly convinced that patients with early AD, care partners and health care systems will benefit from a choice between two alternative treatments with comparable efficacy and safety profiles, but different treatment regimes.

Potential conflicts of interest of individual authors regarding drug development in AD are detailed in the statement below.

## Statement on AI

Generative AI or AI-assisted technology were not used in the preparation of this manuscript.

## CRediT authorship contribution statement

**Frank Jessen:** Writing – review & editing, Writing – original draft, Conceptualization. **Javier Arbizu:** Writing – review & editing, Writing – original draft, Conceptualization. **Mercé Boada:** Writing – review & editing, Writing – original draft, Conceptualization. **Mircea Balasa:** Writing – review & editing, Writing – original draft, Conceptualization. **Karim Bennys:** Writing – review & editing, Data curation, Conceptualization. **Marina Boban:** Writing – review & editing, Writing – original draft, Conceptualization. **Katharina Bürger:** Writing – review & editing, Conceptualization. **Andrea Chincarini:** Writing – review & editing. **Annachiara Cagnin:** Writing – review & editing, Conceptualization. **Peter Paul De Deyn:** Writing – review & editing, Conceptualization. **Emrah Düzel:** Writing – review & editing, Conceptualization. **Sebastiaan Engelborghs:** Writing – review & editing, Conceptualization. **Michael Ewers:** Writing – review & editing, Conceptualization. **Lieza G. Exalto:** Writing – review & editing, Conceptualization. **Wiesje M. van der Flier:** Writing – review & editing, Conceptualization. **Juan Fortea:** Writing – review & editing, Conceptualization. **Kristian Steen Frederiksen:** Writing – review & editing, Conceptualization. **Giovanni B Frisoni:** Writing – review & editing, Conceptualization. **Lutz Frölich:** Writing – review & editing, Conceptualization. **Alejandro J. Garza-Martinez:** Writing – review & editing, Conceptualization. **Timo Grimmer:** Writing – review & editing, Conceptualization. **Bernard Hanseeuw:** Writing – review & editing, Conceptualization. **Jakub Hort:** Writing – review & editing, Conceptualization. **Adrian Ivanoiu:** Writing – review & editing, Conceptualization. **Patrick G Kehoe:** Writing – review & editing, Conceptualization. **Sean P Kennelly:** Writing – review & editing, Conceptualization. **Silke Kern:** Writing – review & editing, Conceptualization. **Stefan Klöppel:** Writing – review & editing, Conceptualization. **Lenka Krajčovičová:** Writing – review & editing, Conceptualization. **Milica G. Kramberger:** Writing – review & editing, Conceptualization. **Bernadette McGuinness:** Writing – review & editing, Conceptualization. **Patrizia Mecocci:** Writing – review & editing, Conceptualization. **Timo Jan Oberstein:** Writing – review & editing, Conceptualization. **Pierre-Jean Ousset:** Writing – review & editing, Conceptualization. **Claire Paquet:** Writing – review & editing, Conceptualization. **Robert Perneczky:** Writing – review & editing, Conceptualization. **Fabrizio Piazza:** Writing – review & editing, Conceptualization. **Domenico Plantone:** Writing – review & editing, Conceptualization. **Innocenzo Rainero:** Writing – review & editing, Conceptualization. **Guillaume Sacco:** Writing – review & editing, Conceptualization. **Eric Salmon:** Writing – review & editing, Conceptualization. **Isabel Santana:** Writing – review & editing, Conceptualization. **Nikolaos Scarmeas:** Writing – review & editing, Conceptualization. **Anja Schneider:** Writing – review & editing, Conceptualization. **Jonathan M Schott:** Writing – review & editing, Conceptualization. **Eino Solje:** Writing – review & editing, Conceptualization. **Elka Stefanova:** Writing – review & editing, Conceptualization. **Elisabeth Stögmann:** Writing – review & editing, Conceptualization. **Mélanie Strauss:** Writing – review & editing, Conceptualization. **Stanislav Sutovsky:** Writing – review & editing, Conceptualization. **Gunhild Waldemar:** Writing – review & editing, Conceptualization. **Bengt Winblad:** Writing – review & editing, Conceptualization.

## Declaration of competing interest

Frank Jessen: FJ has received grants from Roche Pharma and Roche Diagnostics; FJ has received consulting fees from Eli Lilly and Eisai; FJ has received payment or honoraria for lectures, presentations, speakers bureaus, manuscript writing or educational events from Eli Lilly and Eisai; FJ has received payment for expert testimony from Eli Lilly and Eisai; FJ is Chairman of the EADC.

Javier Arbizu: JA has received research funding paid to University of Navarre Clinic from Siemens Healthineers, GE Healthcare, and PET tracer from Life Molecular Imaging; consultancy fees from Novartis and Eli Lilly; and received speaker honoraria from Biogen, Novartis, Life Molecular Imaging, Roche, Novo Nordisk, Siemens Healthineers, GE Healthcare, Zambon.

Mercé Boada: MB has received consulting fees from Grifols, Araclon Biotech, Roche, Biogen, Lilly, Merck, Zambon, Novo Nordisk; MB has received payment or honoraria for lectures, presentations, speakers bureaus, manuscript writing or educational events from Roche, Biogen, Grifols, Nutricia, Araclon Biotech, Servier, Novo Nordisk; MB participated in Data Safety Monitoring Board or Advisory of Board Grifols, Roche, Lilly, Araclon Biotech, Merck, Zambon, Biogen, Novo Nordisk, Bioiberica, Eisai, Servier, Schwabe Pharma, Lighthouse Pharma.

Mircea Balasa: No COI.

Karim Bennys has received grants to his institution from Acadia Pharmaceuticals, Alzheon Inc., Araclon Biotech S.L., AstraZeneca, Biogen, Bristol Myers Squibb, Eisai Inc., Eli Lilly, Genentech, Inc., GlaxoSmithKline, Hoffmann- La Roche, INmune Bio Inc., Janssen Research & Development, Medesis Parma, Merck Sharp & Dohme, Nestlé, Novartis Pharmaceuticals, Novo Nordisk, TauRx Therapeutics Ltd, UCB Biopharma.

Marina Boban has received grants from 4BrainFlames project, EU fund Research (managed by institution), Croatian Science Foundation Research (managed by institution).

Katharina Bürger: Honoraria from Eisai Germany, Lilly Germany, Roche; Travel support by Lilly Germany and Novo Nordisk.

Andrea Chincarin: No COI.

Annachiara Cagnin, AC received speaker honoraria from: PIAM, Eisai, GE, Lilly, Novo Nordisk, Roche.

Peter Paul De Deyn: consulted for Eisai, Biogen, and Perha Pharmaceuticals.

Emrah Düzel: Co-Founder and interim-CEO of neotiv, honoraria for advisory work talks from Roche, Lilly, Eisai, Biogen.

Sebastiaan Engelborghs: SE has received consulting fees from Biogen, Eisai (paid to institution), Icometrix (paid to institution), Janssen, Eli Lilly, Novartis (paid to institution). SE holds patent EP3452830B1 for an assay for the diagnosis of a neurological disease (licensed to ADX Neurosciences NV & Euroimmun Medizinische Labordiagnostika AG). SE is a member of SMB/SAB for EU-H2020 project RECAGE (unpaid) and of the DSMB of PRImus-AD (unpaid).

Michael Ewers: ME received research funding from Eli Lilly.

Lieza G. Exalto: PI at Brain Research Center, however not involved in lecanumab nor donanemab clinical trials.

Wiesje M. van der Flier: WF has been an invited speaker at Biogen MA Inc, Danone, Eisai, WebMD Neurology (Medscape), Novo Nordisk, Springer Healthcare, European Brain Council. WF is consultant to Oxford Health Policy Forum CIC, Roche, Biogen MA Inc, Eisai, Eli-Lilly, Owkin France. WF participated in advisory boards of Biogen MA Inc, Roche, and Eli Lilly. WF is member of the steering committee of phase 3 EVOKE/EVOKE+ studies (Novo Nordisk). WF is member of the steering committee op phase 3 Trontinemab study (Roche). All funding is paid to her institution.

WF is member of the steering committee of PAVE, and Think Brain Health. WF is chair of the Scientific Leadership Group of InRAD. WF was associate editor of Alzheimer, Research & Therapy in 2020/2021. WF is associate editor at Brain. WF is member of Supervisory Board (Raad van Toezicht) Trimbos Instituut.

Juan Fortea: JF reported serving on the advisory boards, adjudication committees, or speaker honoraria from AC Immune, Adamed, Alzheon, Biogen, Eisai, Esteve, Fujirebio, Ionis, Laboratorios Carnot, Life Molecular Imaging, Lilly, Lundbeck, Novo Nordisk, Perha, Roche, Zambón, Spanish Neurological Society, T21 Research Society, Lumind foundation, Jérôme-Lejeune Foundation, Alzheimer’s Association, National Institutes of Health USA, and Instituto de Salud Carlos III. JF reports holding a patent for markers of synaptopathy in neurodegenerative disease (licensed to ADx, EPI8382175.0). No other competing interests were reported.

Kristian Steen Frederiksen: KF has participated in advisory boards/consulted for Novo Nordisk, Roche Diagnostics, Eisai/Bioarctic, Eli Lilly (paid to institution), honoraria for lectures from Eisai/Bioarctic, Nono Nordisk, Eli Lilly (paid to institution), Editor-in-Chief, Alzheimer´s Research & Therapy (Personal renumeration form Springer Nature).

Giovanni B Frisoni: GBF has received funding through the University of Geneva or Geneva University Hospitals: for IISSs from ROCHE Pharmaceuticals OM Pharma EISAI Pharmaceuticals Biogen Pharmaceuticals and Novo Nordisk. He has received payment or honoraria for lectures, presentations, speakers bureaus, manuscript writing, or educational events from: Biogen, Roche, Novo Nordisk, GE HealthCare. All through institution.

Lutz Frölich: Honoraria from Biogen, BioVie, Eisai, Eli Lilly, FOMF, Araclon/Grifols, Janssen Cilag, Medical Tribune, Medfora, Neurimmune, Noselab, Novo Nordisk, Roche, TauRX, Schwabe, StreamUp, Avanir/Otsuka, PharmatrophiX, Charité Berlin, Neuroscios, Vivoryon; Research support: Roche.

Alejandro J. Garza-Martinez: No COI.

Timo Grimmer: TG reported receiving consulting fees from Acumen, Advantage Ther, Alector, Anavex, Biogen, BMS; Cogthera, Eisai, Eli Lilly, Functional Neuromod., Grifols, Janssen, Neurimmune, Noselab, Novo Nordisk, Roche Diagnostics, and Roche Pharma; lecture fees from Anavex, Cogthera, Eisai, Eli Lilly, FEO, Grifols, Pfizer, Roche Pharma, Schwabe, and Synlab; and has received grants to his institution from Biogen, Eisai and Eli Lilly.

Bernard Hanseeuw: consulted for Eisai, Biogen, and Roche.

Jakub Hort: consulted for Eisai, Biogen, Roche and Eli Lilly, speakers honoraria from Eli Lilly, Schwabe, stock options in Alzheon.

Adrian Ivanoiu: No COI.

Patrick G Kehoe: has received Research Grants from BRACE, Alzheimer’s Brain Banks UK, the Bright Focus Foundation and R01AG074258 NIH (NIA); Payments made to the Institution for Biomedical related research projects on post mortem brain tissue unrelated to these drugs.

Sean P Kennelly: SPK has received payment or honoraria for lectures, presentations, speakers bureaus, manuscript writing or educational events from Roche, Novo Nordisk, Eisai, Biogen and Nutricia; SPK has received support for attending meetings and/or travel from Roche and Nutricia.

Silke Kern: SK has served at scientific advisory boards, speaker and / or as consultant for Roche, Eli Lilly, Geras Solutions, Optoceutics, Biogen, Eisai, Merry Life, Triolab, Novo Nordisk and Bioarctic.

Stefan Klöppel: SK has received various research grants but not related data reported; SK has received consulting fees from OM Pharma (Participantion dementia summit), SK has received payment or honoraria for lectures from OM Pharma (personal payment); participation on Advisory boards 2023: Advisory Board Alzheimer's Disease - Eisai Switzerland 2024: Advisory Board Rexulti AA – Lundbeck 2025: Advisory Board Alzheimer’s Disease- Eisai (personal payment).

Lenka Krajčovičová: No COI.

Milica G. Kramberger: MGK has received consulting fees from Lilly; MGKhas received payment or honoraria for lectures, presentations, speakers bureaus, manuscript writing or educational events from Lilly; MGK has participated in a Data Safety Monitoring Board or Advisory Board for Lilly.

Bernadette McGuinness: has received consulting fees from Biogen, Lilly, Eisai (payment made personally); has received payment or honoraria for Chair for Lilly sponsored event at British Geriatrics Society meeting 2024 (payment made personally).

Patrizia Mecocci: PM has received grants or contracts from HORIZON-JU-RIA Program AD-Riddle, Horizon 2020 Lethe; PM has received payment or honoraria for lectures, presentations, speakers bureaus, manuscript writing or educational events from Piam, Angelini, Epitech; PM has participated in a Data Safety Monitoring Board or Advisory Board Primus; PM is Treasurer Italian Academy of Geriatrics, Member Board of Directors SIGOT.

Timo Jan Oberstein: TJO received lecture honoraria from: Biogen, Lilly, Siemens Healthineers.

Pierre-Jean Ousset: has received consulting fees from Eli Lilly’s, EISAI’s, International Board and national board.

Claire Paquet: CP has received consulting fees from Eli Lilly’s, EISAI’s, Roche’s, Novo Nordisk’s, UCB’s International Board and national board; CP has received payment or honoraria for lectures for Eli Lilly and EISAI; CP has received payment for expert testimony from Eli Lilly (national testimony with patient in April 2025).

Robert Perneczky: RP has received honoraria for advisory boards and speaker engagements from Roche, EISAI, Eli Lilly, Biogen, Janssen-Cilag, Astra Zeneca, Schwabe, Grifols, Novo Nordisk and Tabuk.

Fabrizio Piazza: Consulting fees, advisory board fees, and educational activities, paid to the University of Milano Bicocca, with Alnylam Pharmaceuticals, Roche, Biogen, Alector, Lilly, Araclon, Eisai. FP is the inventor, without ownership, of the amyloid-beta antibody ultrasensitive assay.

Domenico Plantone: DP received travel reimbursments from Fujirebio Italia srl.

Innocenzo Rainero: IR has recevied consulting honoraria from Eli Lilly.

Guillaume Sacco: has received payment or honoraria for lectures, presentations, speakers bureaus, manuscript writing or educational events from EISAI and Biogen, has received support for attending meetings and/or travel from Biogen and BMS.

Eric Salmon: Advisory Board for Biogen, Eisai, Will Pharma.

Isabel Santana: IS has received funding through the University of Coimbra: for IISSs from FCT (National Agency for Research), ROCHE Pharmaceuticals OM Pharma Biogen Pharmaceuticals . She has received payment or honoraria for lectures, presentations, speakers or bureaus, from: Biogen, Lilly, EISAI, Novo Nordisk.

Nikolaos Scarmeas: Novo Nordisk - Local PI of recruiting site for multinational, multicenter industry sponsored phase III treatment trial for Alzheimer's disease - funding to institution.

Anja Schneider: Research Grant from Eisai (2020-2023), SAB for Biogen CELIA study, received honoraria from Eisai and Biogen.

Jonathan M Schott: JMS has received research funding and PET tracer from AVID Radiopharmaceuticals (a wholly owned subsidiary of Eli Lilly) and Alliance Medical; has consulted for Roche, Eli Lilly, Biogen, MSD and GE; and received royalties from Oxford University Press and Henry Stewart Talks. He is an NIHR Senior Investigator and Chief Medical Officer for Alzheimer’s Research UK.

Eino Solje: E. Solje has served on the advisory board of Novartis, EISAI, Lilly and Roche, served as a consult for Novo Nordisk, BioaArctic, EISAI, Lilly and Roche and received honoraria from for lectures from Lundbeck, BioaArctic and Roche and travel support from Lilly.

Elka Stefanova: ES has received grants from AACSFD-17–533520, IGPCC-B-1262787, National Grant Republic of Serbia (451–03–66/2024-03/200110) (payment made to the institution); ES has received support for attending meetings and/or travel from Teva, Goodwil Pharma, Krka (payment made to the institution).

Elisabeth Stögmann`s institution (Medical University of Vienna, Austria) received financial support by research grants for research in AD: Eisai, EU Horizon 2020, Austrian Research Promotion Agency).

Elisabeth Stögmann`s institution (Medical University of Vienna, Austria) received financial support for participation in clinical trials from Biogen, Novo Nordisk and Roche Diagnostics.

Elisabeth Stögmann received honoraria (consultations, lectures, speakers bureaus, educational events, advisory boards) from pharmaceutical companies: Biogen, Roche, Novo Nordisk, Eisai, Novartis, Sanofi and Lilly.

Elisabeth Stögmann held leadership in scientific societies: Austrian Alzheimer Association.

Mélanie Strauss: consulted for Eisai.

Stanislav Sutovsky No COI.

Gunhild Waldemar: GW reported no conflicts of interest.

Bengt Winblad: BW serves as member of Scientific Advisory Boards (SABs) for Alzinova, Axon Neuroscience, Artery Therapeutics, Phanes Biotech and is a member of the DSMB for Primus-AD. BW has stocks in Artery Therapeutics and AlzeCure.
